# OmpA controls order in the outer membrane and shares the mechanical load

**DOI:** 10.1073/pnas.2416426121

**Published:** 2024-12-04

**Authors:** Georgina Benn, Carolina Borrelli, Dheeraj Prakaash, Alex N. T. Johnson, Vincent A. Fideli, Tahj Starr, Dylan Fitzmaurice, Ashton N. Combs, Martin Wühr, Enrique R. Rojas, Syma Khalid, Bart W. Hoogenboom, Thomas J. Silhavy

**Affiliations:** ^a^Department of Molecular Biology, Princeton University, Princeton, NJ 08540; ^b^London Centre for Nanotechnology, University College London, London WC1H 0AH, United Kingdom; ^c^Department of Physics & Astronomy, University College London, London WC1E 6BT, United Kingdom; ^d^Centre for Bacterial Resistance Biology, Imperial College London, London SW7 2AZ, United Kingdom; ^e^Department of Biochemistry, University of Oxford, Oxford OX1 3QU, United Kingdom; ^f^Department of Chemical and Biological Engineering, Princeton University, Princeton, NJ 08540; ^g^Lewis-Sigler Institute for Integrative Genomics, Princeton University, Princeton, NJ 08540; ^h^Department of Cell and Developmental Biology, University College London, London WC1E 6BT, United Kingdom; ^i^Department of Biology, New York University, New York, NY 10003

**Keywords:** outer membrane, atomic force microscopy, membrane biophysics, membrane organisation

## Abstract

OmpA, a predominant outer membrane (OM) protein in *Escherichia coli*, affects virulence, adhesion, and bacterial OM integrity. However, despite more than 50 y of research, the molecular basis for the role of OmpA has remained elusive. In this study, we demonstrate that OmpA organizes the OM protein lattice and mechanically connects it to the cell wall (CW). Using gene fusions, atomic force microscopy, simulations, and microfluidics, we show that the β-barrel domain of OmpA is critical for maintaining the permeability barrier, but both the β-barrel and CW–binding domains are necessary to enhance the cell envelope’s strength. OmpA integrates the compressive properties of the OM protein lattice with the tensile strength of the CW, forming a mechanically robust composite that increases overall integrity. This coupling likely underpins the ability of the entire envelope to function as a cohesive, resilient structure, critical for the survival of bacteria.

## Significance

The high resistance of Gram-negative bacteria to antibiotics is largely due to the protein-rich outer membrane (OM) that surrounds these cells. One of the most abundant proteins in the OM is OmpA, with similar proteins found in almost all Gram-negative species. Here, we show the role of OmpA is to order the immobile lattice of other OM proteins and directly couple this lattice with the underlying cell wall (CW). This OmpA-based connection allows the CW and OM protein lattice to behave as a composite material, far stronger than they would be without a direct connection. The high mechanical loads the cell envelope is subjected to are therefore distributed across these layers by OmpA, increasing the survival and virulence of bacteria.

Antibiotic resistance in Gram-negative (diderm) bacteria, such as *Escherichia coli*, is a serious and increasing healthcare burden (1). Much of this resistance is attributed to the impermeable outer membrane (OM) that surrounds cells, preventing antibiotic access to intracellular targets (2). The OM can also be disrupted, allowing drugs to bypass the permeability barrier. However, it remains unclear why this barrier is essential for viability (3), and how its connection to the underlying cell affects survival.

Below the OM is the periplasmic space: a dense, aqueous compartment with high concentrations of proteins including chaperones and protective enzymes, like phosphatases and nucleases, that would be toxic in the cytoplasm (4, 5). The periplasm also includes the cell wall (CW) which is often only a few nanometers thick in diderms but can be up to hundreds of nanometers thick in some of these species (6). The CW is made up of extended glycan strands linked by peptide bonds and has long been known to maintain cell shape, to resist turgor pressure, and to provide mechanical strength to the cell (7–9). The inner membrane then separates the periplasm and cytoplasm; this is a fluid phospholipid bilayer with α-helical transmembrane proteins and acyl-anchored lipoproteins (10). The OM, CW, and inner membrane layers form the basis of the diderm envelope, but the structural diversity of these layers across the bacterial kingdom is vast.

*E. coli* OM structure is by far the most studied of any organism. Unlike traditional bilayer membranes, it has an asymmetric lipid bilayer with a very high protein content. Phospholipids are excluded from the outer leaflet of the OM, which is instead occupied by lipopolysaccharide (LPS) (5). Phosphates in the core of LPS are strongly bridged by divalent cations, and polysaccharides protrude away from the cell to maximize intermolecular contacts between neighboring chains (11). These tight interactions make the OM virtually impermeable to molecules that cannot enter via OM proteins (OMPs) (12).

Almost all OMPs contain transmembrane β-barrels and can be highly abundant, with some at more than 100,000 copies per cell, including OmpA, OmpC, and OmpF in *E. coli* (5, 13). These latter two OMPs are porins of 16-stranded barrels that form trimers and allow small, hydrophilic molecules to diffuse freely across the membrane. The trimeric porins are packed into the OM in a virtually immobile, imperfect hexagonal lattice that spans the whole cell (14). This lattice is fused together by LPS molecules, often with only one LPS molecule between neighboring OMPs (15). This OMP-LPS-OMP arrangement allows strong, nonspecific interactions between the diverse proteins embedded in the lattice (15).

While it is unlikely to be completely universal, the existence of a β-barrel protein lattice appears widespread across many clades: Dense, regular lattices have been seen in the OM of Terrabacteria (16), Gracilicutes (14, 17–19), and are even readily induced in mitochondrial OM (20, 21). Another recurring feature of bacterial OMs is tethering to the underlying CW. In Terrabacteria, this is largely performed by OmpM or OmpM-homologues, which all have a CW-binding S-layer homology domain attached to an OM β-barrel. The importance of these tethers is reflected by their presence in all diderm phyla of the clade, often with multiple copies (10). In *E. coli* and other Proteobacteria, tethering is mostly done by three major proteins: Pal, Lpp, and OmpA (5). Pal and Lpp are both lipoproteins, anchored to the inner leaflet of the OM via three acyl chains attached to an N-terminal cysteine (22). The role of Pal is predominantly in cell division, where it pulls the OM down at the division septum and removal of *pal* significantly decreases cellular fitness (23). Lpp is the most abundant protein in *E. coli* (5) with a rigid coiled-coil structure that covalently anchors the OM to the CW and dictates the periplasmic width (24).

The structure of OmpA is an 8-stranded β-barrel, attached via a flexible linker, to a periplasmic C-terminal domain that noncovalently binds the CW (25). However, pinning down the precise role of OmpA has proved difficult, despite it being one of the first proteins identified in bacteria and having a known importance in virulence, adhesion, and OM integrity (26–28).

As our appreciation of the diversity of diderm envelopes has improved, the known roles of the *E. coli* OM have also expanded. As well as protecting cells from harmful molecules in the environment, it has recently been shown that the OM significantly contributes to cell strength and shape (29, 30). However, it is not known which OM components contribute to these roles, how this is distributed to the rest of the cell envelope, or how universal these roles are. Here, we dissect the role OmpA plays in OM permeability, strength, and lattice integrity using genetics, microfluidics, coarse-grain simulations, and atomic force microscopy (AFM). We show that beyond tethering the OM, OmpA acts as a static fencepost to order surrounding OMPs into an immobile lattice and to anchor that lattice to the CW. This directly couples the OMP lattice and CW to produce a mechanically strong composite that is far more robust than the two layers separately.

## Results

### OmpA Contributes to OM Integrity.

**Fig. 1.**
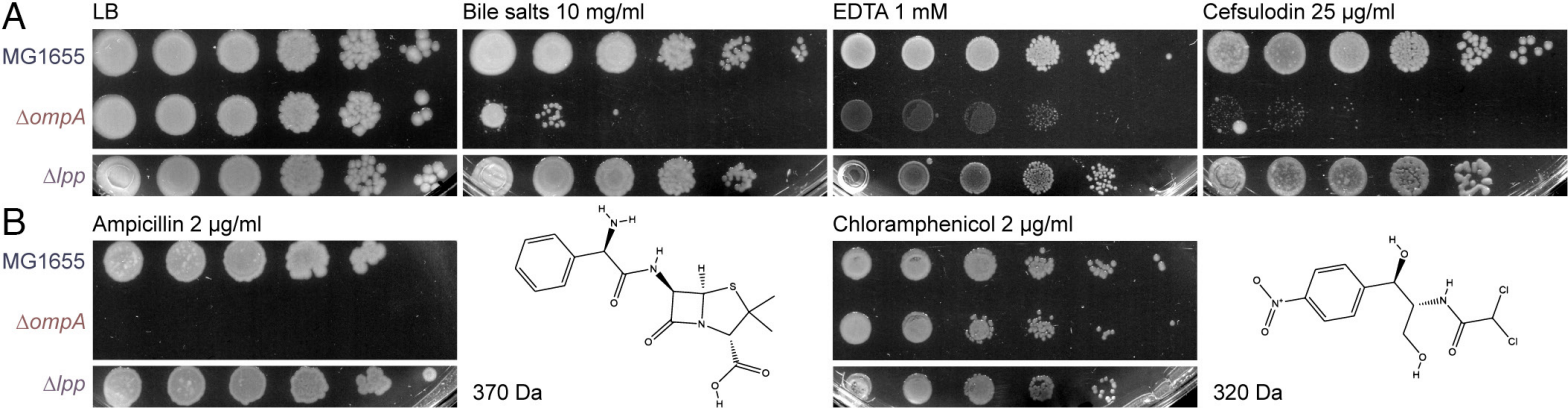
The efficiency of plating assays show that OmpA contributes to OM integrity. (*A* and *B*) Plating efficiency of MG1655 WT, ∆*ompA,* and ∆*lpp* cells on LB agar and LB agar supplemented with indicated antimicrobials. Dilutions from left to right are 10^0 to^ 10^−5^. (*B*) The similar structures of ampicillin and chloramphenicol are indicated to the right, with their similar molecular weights.

The overlapping roles of *lpp* and *ompA* are evidenced by OM shedding in *E. coli* lacking both these genes, compared to little effect if only one is lost (31). However, ∆*lpp* and ∆*ompA* cells have distinct permeability phenotypes. In particular, and unlike ∆*lpp* cells, ∆*ompA* cells are sensitive to antimicrobials that affect the OM outer leaflet, like bile salts and EDTA (12), or those that affect CW synthesis (Fig. 1 *A* and *B*). The enhanced sensitivity to CW-disrupting drugs is particularly striking. Where ∆*lpp* cells grow at similar levels to the wild type (WT) when exposed to low concentrations of the β-lactams ampicillin and cefsulodin, ∆*ompA* cells do not survive (Fig. 1 *A* and *B*). Importantly, sensitivity to the similarly sized, porin-crossing translation inhibitor, chloramphenicol, is unaffected, indicating that the sensitivity to the β-lactams is not due to a general increase in OM permeability (Fig. 1*B*). These observations suggest the main role of OmpA is different from Lpp, where a lack of OmpA makes the outer leaflet easier to disrupt and cells more sensitive to changes in CW integrity.

### A β-Barrel Is Essential for OmpA Function.

To probe the importance of OmpA, we first considered its major differences from Lpp. They differ in that β-barrel OMPs, like OmpA, are notoriously static in the membrane (14, 32), whereas inner leaflet-anchored lipoproteins are more mobile when not restricted by binding to the CW (23), suggesting the N terminus of a lipoprotein can diffuse in its local phospholipid environment. A key difference between lipoproteins and OMPs is that inner leaflet lipoproteins, like Pal and Lpp, cannot interact with LPS since they cannot access the outer leaflet. Third, OMPs occupy volume in the outer leaflet. This is particularly true of the abundant OmpA. Since mass spectrometry detects no change in expression of any other OMPs upon deletion of *ompA*, and western blotting showed no difference in trimeric porin abundance (*SI Appendix*, Fig. S1), it appears that the deletion of *ompA* results in significant loss of volume occupied by OMPs. The absence of change in expression of other OMPs also suggests that neither the Cpx nor σ^E^ stress responses are activated in the ∆*ompA* cells (33).

**Fig. 2.**
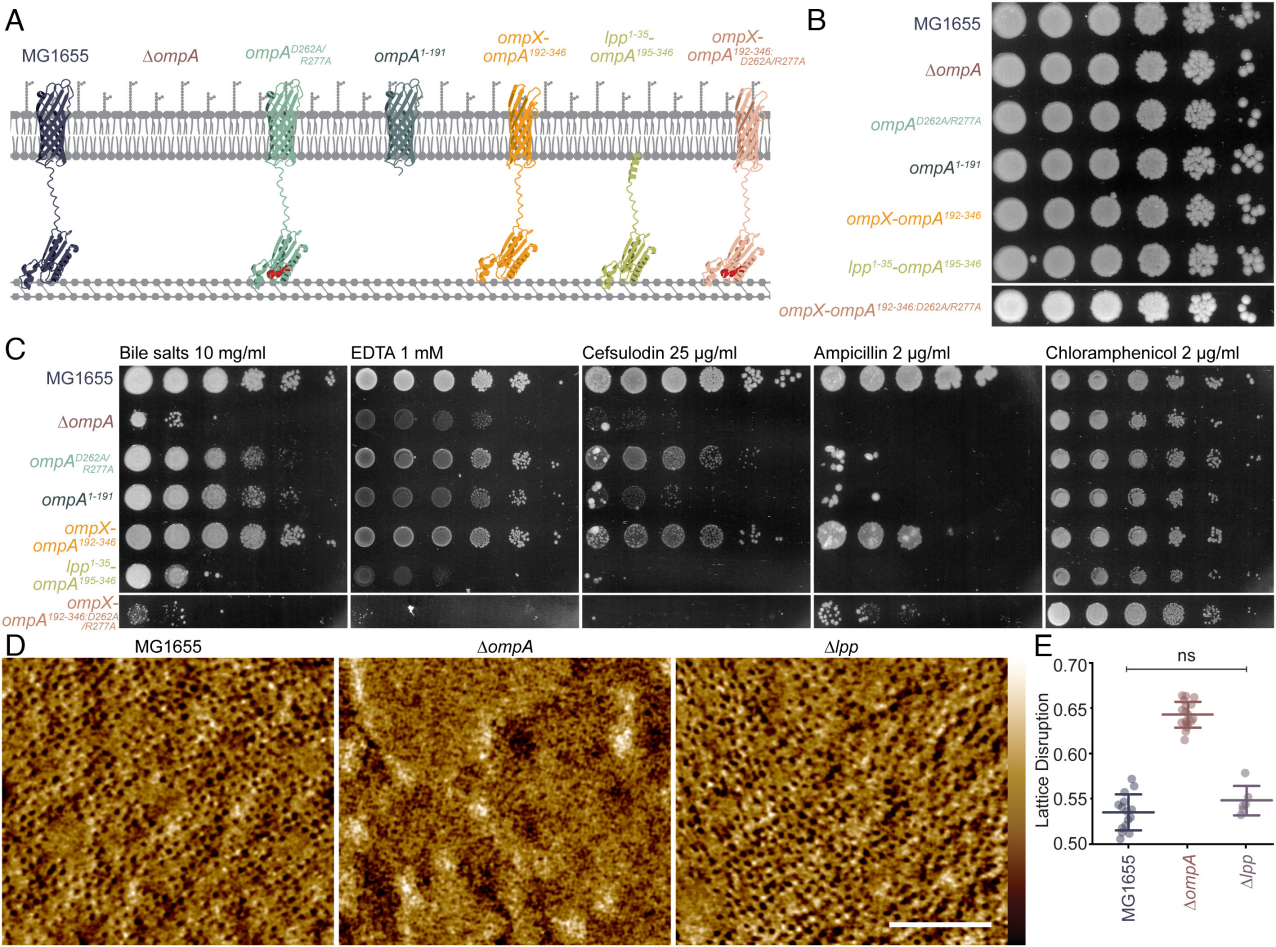
OmpA constructs and their impact on the efficiency of plating show that the β-barrel is particularly important for function, and AFM shows that removal of OmpA compromises OMP lattice order. (*A*) Schematic representations of the seven main strains used throughout the study. MG1655 has full-length OmpA, with an 8-stranded β-barrel domain, linker, and C-terminal CW binding domain. To investigate the importance of CW binding, this was reduced by mutating two residues important for binding (*ompA^D262A/R277A^*) or binding was abolished by complete removal of the C-terminal domain (*ompA^1-191^*). To investigate the importance of the β-barrel, the OmpA β-barrel was replaced with that of OmpX (*ompX-ompA^192-346^*) or by the beginning of Lpp (*lpp^1-35^-ompA^195-346^*). To test the relative importance of CW binding in *ompX-ompA^192-346^,* the C-terminal domain was also mutated (*ompX-ompA^192-346: D262A/R277A^*). All constructs were inserted into the genome at the *ompA* locus and were well expressed, as detected by western blotting (*SI Appendix*, Fig. S2). (*B* and *C*) Plating efficiency of MG1655 WT and indicated strains on (*B*) LB agar and (*C*) LB agar supplemented with indicated antimicrobials. *ompA^D262A/R277A^*, *ompA^1-191^*, and *ompX-ompA^192-346^* survive well on bile and EDTA plates and have intermediate growth on cefsulodin and ampicillin. Like ∆*ompA* cells, *lpp^1-35^-ompA^195-346^* and *ompX-ompA^192-346:D262A/R277A^* grow poorly in the presence of bile, EDTA, cefsulodin, or ampicillin. Dilutions from left to right are 10^0 to^ 10^−5^. (*D*) Live cell AFM phase images of the WT, ∆*ompA,* and ∆*lpp* OMs show that the OMP lattice is abolished without OmpA. (Scale bar, 100 nm.) Color scales are 0.9, 0.8, and 0.6 deg. Color range is shown to the *Right* of panel A. (*E*) Quantification of lattice disruption shows that deletion of *ompA* significantly increases lattice disruption compared to the WT, whereas removing *lpp* has no effect. Center lines are means, and error bars are SD. ns = (*P* > 0.15) by the two-sample *t* test.

Considering these differences, we constructed five versions of OmpA (Fig. 2*A*) and assessed their function by plating efficiency. Compared to ∆*ompA*, introduction of OmpA with reduced or absent CW binding, by point mutation (Fig. 2*A*; *ompA^D262A/R277A^*) and truncation (Fig. 2*A*; *ompA^1-191^*), improves EDTA and bile salts resistance, and slightly improves resistance to β-lactams, suggesting CW binding is somewhat important for function, but not essential (Fig. 2*C*). Strikingly, replacing the β-barrel of OmpA with the beginning of Lpp (Fig. 2*A*; *lpp^1-35^-ompA^195-346^*), thus anchoring the periplasmic domain to the OM only via the inner leaflet, completely abolishes OmpA function compared to the WT. This conclusively shows that OmpA does not merely tether the OM to the CW, as has been previously proposed (10). Instead, it indicates that the β-barrel of OmpA is particularly important for function (Fig. 2*C*).

To determine whether the importance of the β-barrel of OmpA is due to specific interactions, we next replaced the barrel of OmpA with OmpX (34) (Fig. 2*A*; *ompX-ompA^192-346^*). Like OmpA, OmpX is an 8-stranded β-barrel but it has no sequence similarity to OmpA and has different LPS binding patterns (35). Therefore, OmpX-OmpA^192-346^ will occupy a similar volume in the OM to OmpA, but interact with surrounding LPS and OMPs with different orientations and affinities, thus disrupting the OMP-LPS lattice.

Surprisingly, this *ompX-ompA^192-346^* construct rescues sensitivity to outer leaflet disruption well and to CW disruption somewhat (Fig. 2*C*). This could suggest that the contribution of OmpA to the impermeability barrier does not strongly depend on specific barrel interactions. However, *ompX-ompA^192-346^* does not completely mimic the WT, with lower resistance to ampicillin and cefsulodin, although this may be due to slightly lower levels than the highly abundant OmpA (*SI Appendix*, Fig. S2). Furthermore, *ompX-ompA^192-346:D262A/R277A^* cells, where CW binding is reduced (Fig. 2*A*), or cells in which *ompA* is replaced with only *ompX* behave like the ∆*ompA* deletion (Fig. 2*C* and *SI Appendix*, Fig. S3). First, this suggests the decrease in permeability is not due to a general increase in OM volume occupied by OMPs. Second, this shows that the β-barrel of OmpA is the most essential domain for function, that there is some sequence specificity, and that the CW binding C-terminal domain additively improves function.

### Removal of OmpA Abolishes the OMP Lattice.

To investigate why removing OmpA decreases OM integrity, we next looked at the OM in vivo using high-resolution AFM on the surface of live, growing bacteria. As expected, AFM shows that the OM of wild-type cells is filled with an imperfect hexagonal lattice of trimeric porins, with other OMPs embedded between them (14, 15), and each image has well-resolved trimers (Fig. 2*D*). However, removal of OmpA dramatically abolishes the visibility of the porin lattice by AFM, despite no significant change in OMP composition, except loss of OmpA (*SI Appendix*, Fig. S1).

AFM is a surface scanning technique which requires a relatively static sample for two reasons: so that the imaged object is in the same position in subsequent scan lines and so that the AFM tip does not nudge highly mobile objects around while it is gently scanning. Therefore, by AFM, resolution tends to degrade with increasing mobility of the objects under study (36). The inability to image trimeric porins in ∆*ompA* mutants by AFM therefore suggests these proteins are less ordered, potentially due to increased local mobility, since they are not corralled by OmpA. For comparison between strains, lattice disruption was quantified by the inverse of the confidence with which OMP trimers could be resolved by AFM. Briefly, this quantification used a cross correlation to compare each possible pore of an image to an ideal pore. When lattice disruption is low, the OMP trimers are well resolved (see *Materials and Methods* and *SI Appendix*, Fig. S4). This clearly shows that the OMP lattice is disrupted in ∆*ompA* cells (Fig. 2*E*).

As well as a remarkable increase in lattice disruption, the OMs of ∆*ompA* cells also have large, flat plateaus sitting 1 to 2 nm above the rest of the membrane (*SI Appendix*, Fig. S5), suggesting serious defects in OM integrity. However, the low resolution of the OM in these cells precludes our ability to quantify these defects. Previous research has also shown that the removal of OmpA leads to a decrease in cell stiffness (29) so it was possible that the inability to image the OMP lattice was due to an overall softer cell. However, the resolution of the lattice in similarly soft cells, like ∆*lpp* (29), was unaffected (Fig. 2 *D* and *E*), showing that the low resolution of ∆*ompA* cells is not due to general softening of the cell. This also shows that a general reduction in OM tethering does not disrupt the lattice, and this is specific to removal of OmpA.

These observations are particularly notable considering previous research showing the OMP lattice to be remarkably tolerant of changes to OM composition: OMP lattice spacing and packing is unchanged by decreased LPS content or introduction of outer leaflet phospholipids, and trimeric OMPs remain immobile when overall porin levels are decreased (14). We therefore hypothesized that OmpA has a distinct role in OMP lattice order, using the CW binding domain to stay in place and the β-barrel to interact with surrounding OMPs via LPS.

### OmpA Controls OM Order.

**Fig. 3.**
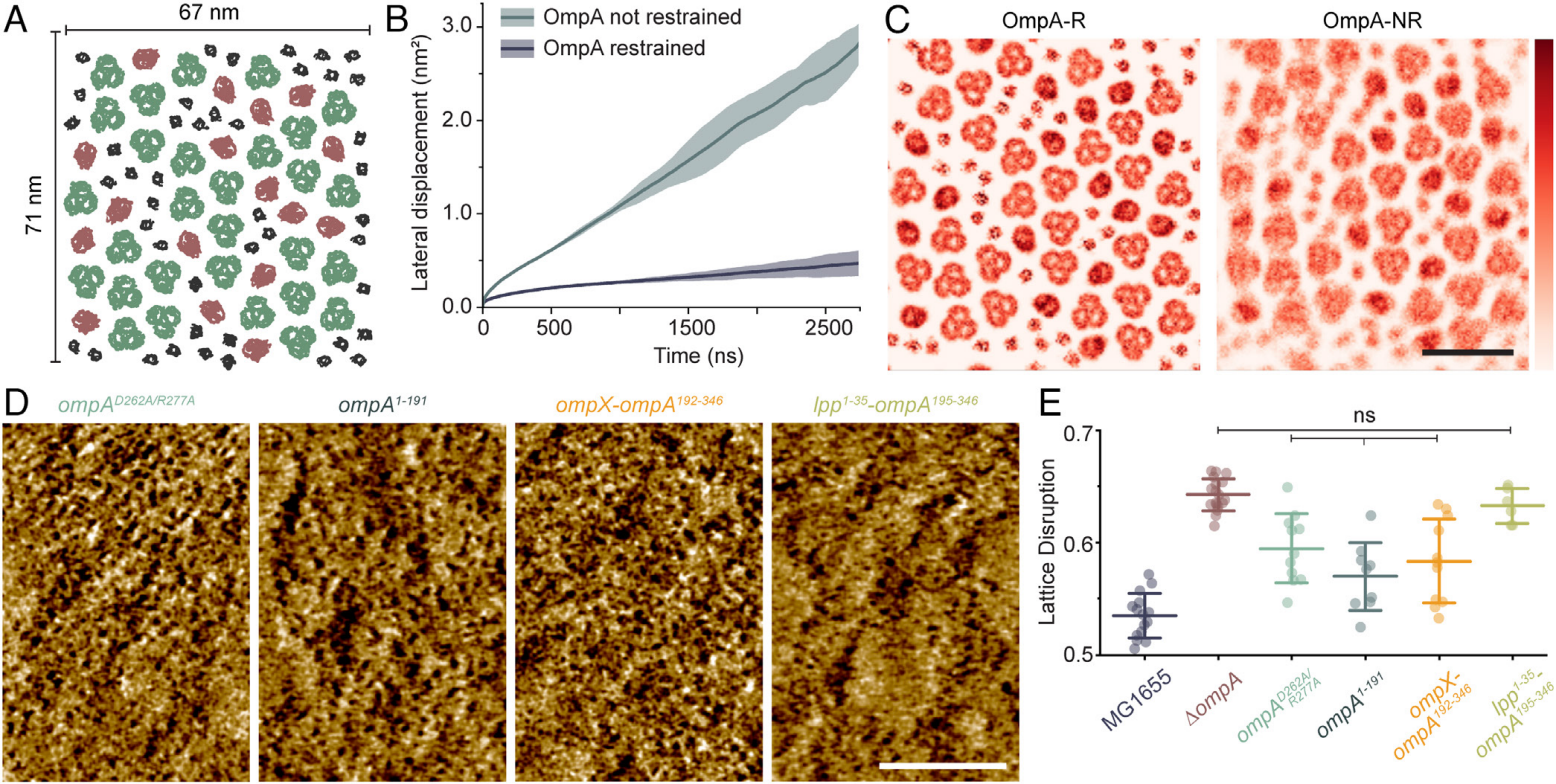
Simulations and AFM show that a CW–tethered OmpA can order surrounding OMPs. (*A*) Top–down view of the simulation setup. 29 copies of OmpF trimers (green), 17 copies of FhuA (red), and 44 OmpA (black) are arranged in an imperfect hexagonal lattice. ReLPS molecules are not shown, for clarity. (*B*) Lateral displacement of OmpF and FhuA proteins versus time, with corresponding slope. For each condition, data from three individual simulations were taken to obtain averages (shown as lines) and SD (shaded regions). In OmpA-NR conditions, surrounding proteins are significantly more mobile. (*C*) Density maps showing the lateral displacement of OMPs over the 2.75 µs-long production simulation when OmpA is restrained (OmpA-R) and not restrained (OmpA-NR). The increased mobility is seen as a blurring of proteins, but each protein stays approximately in its place in the lattice. (Scale bar, 20 nm.) Color density scale, shown on the right, is from 0 to 6. (*D*) Live cell AFM phase images of indicated strains show that the OMP lattice is better resolved in *ompA^D262A/R277A^*, *ompA^1-191^*, and *ompX-ompA^192-346^* strains than the deletion, but *lpp^1-35^-ompA^195-346^* lattice disruption is not improved. (Scale bar, 100 nm.) Color scales are 0.25, 0.8, 0.4, and 0.9 deg. (*E*) Quantification of lattice disruption shows that *ompA^D262A/R277A^, ompA^1-191^*, and *ompX-ompA^192-346^* all have significantly lower lattice disruption than ∆*ompA* but are still significantly different from the WT. *lpp^1-35^-ompA^195-346^* cells have lattice disruption equal to ∆*ompA*. Center lines are means, and error bars are SD. ns = (*P* > 0.08) by the two-sample *t* test.

To initially investigate whether OmpA can stabilize other OMPs, we performed coarse-grained (Martini 2) (37) simulations of the OM. A bilayer with an outer leaflet of fast deep rough LPS (ReLPS) (38) and an inner leaflet of phosphoethanolamine, palmitoylphosphatidylglycerol, and cardiolipin was embedded with OmpF, FhuA, and OmpA arranged in a roughly hexagonal lattice with 9 nm spacing, avoiding LPS-only regions around OMPs, to mimic the dense arrangement of OMPs in the OM (14, 15) (Fig. 3*A*). To avoid differences in protein content, we compared the lattice with or without OmpA tethering to the CW, rather than with or without OmpA altogether, mimicking wild-type OmpA vs OmpA^1-191^ (Fig. 2*A*). This was tested using restrained (OmpA-R) and not restrained (OmpA-NR), where OmpA-R is fixed in the starting position and OmpA-NR is free to diffuse in the membrane like other OMPs (39, 40). By plotting the mean square displacement of OmpF and FhuA along the plane of the membrane versus time, we see that the lateral displacement of OMPs is significantly reduced by just restraining the position of OmpA, leading to an eightfold decrease in diffusion (Fig. 3*B*). Furthermore, density maps of the positions of OmpA, OmpF, and FhuA over the simulation duration suggest that while OMPs diffuse locally with OmpA-NR, they stay in a roughly hexagonal arrangement, suggesting the OMP lattice is still present when OmpA function is reduced, but the disruption makes OMPs more locally mobile (Fig. 3*C*).

To investigate these effects in vivo, we next turned to AFM. If the lattice is being ordered by OmpA, as predicted, we would expect OmpX-OmpA^192-346^, OmpA^D262A/R277A^, and OmpA^1-191^ all to perform well, since they can interact with surrounding OMPs, but Lpp ^1-35^-OmpA^195-346^ to perform poorly. Indeed, AFM shows that *lpp ^1-35^-ompA^195-346^* cells have similar disruption to ∆*ompA*. Furthermore, a significant decrease in lattice disruption is seen for *ompX-ompA^192-346^*, *ompA^D262A/R277A^*, and *ompA^1-191^* OMs compared to ∆*ompA*, although still significantly higher than the WT (Fig. 3 *D* and *E*). AFM images of *ompA^1-191^* OMs were surprisingly similar to density maps from simulations, where each pore can be seen, but the resolution is significantly worse, suggesting OMPs are more mobile, but staying approximately in place (Fig. 3 *C* and *D*).

The good performance of OmpX-OmpA^192-346^ suggests that despite improper barrel interactions, the stability offered by CW binding improves OmpA function. Similarly, the ability of OmpA^1-191^ to provide lattice order without CW binding suggests that interacting with surrounding LPS or OMPs is important. Overall, this evidence suggests that for OmpA to perform any function, the barrel of OmpA must either make sequence-dependent interactions in the outer leaflet or remain fixed in place by the C-terminal domain; and for full function both must occur. We therefore conclude that OmpA orders the OM by remaining immobile and strongly interacting with surrounding OMPs or LPS.

### Cell Envelope Stiffness Requires a Fully Functional OmpA.

**Fig. 4.**
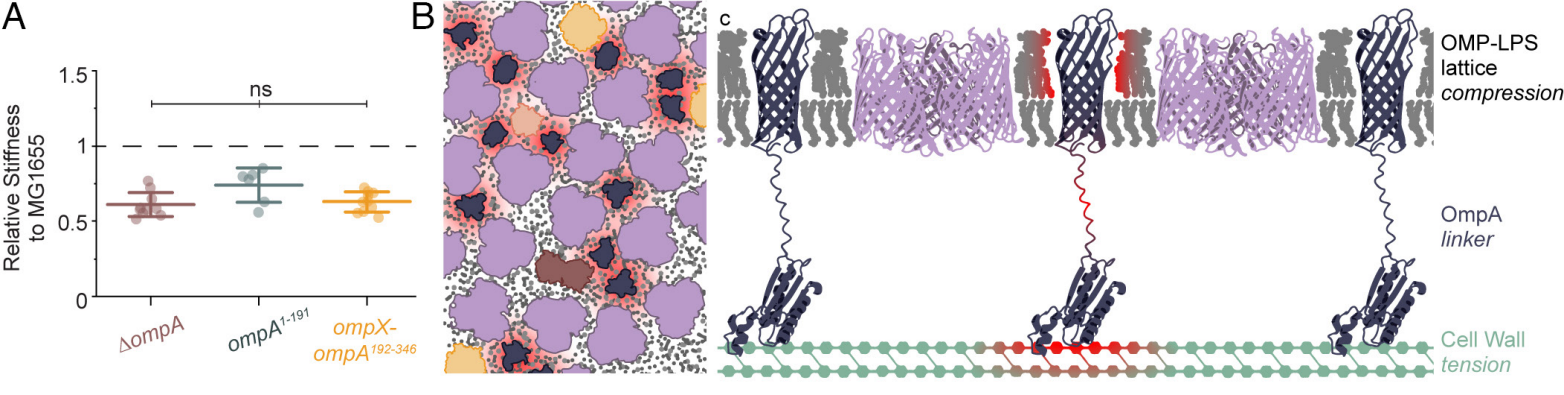
Only full-length OmpA contributes to cell strength. OmpA orders the OMP lattice and connects the OM and CW to form a mechanically strong composite. (*A*) Osmotic force extension was used to calculate the relative stiffness of ∆*ompA*, *ompA^1-191^*, and *ompX-ompA^192-346^* cell envelopes, normalized to the WT (MG1655). This shows that all three strains are significantly softer than the WT (*P* < 0.003 by the one-sided *t* test). Center lines are means, and error bars are SD. ns = (*P* > 0.15) by the two-sample *t* test. (*B*) Schematic of predicted OM organization. The hexagonal lattice of trimeric porins (purple) is embedded with other OMPs (orange and brown), leaving gaps that are filled by the abundant OmpA (blue) which interacts with surrounding OMPs (red regions) while remaining in place, maintaining an ordered lattice. (*C*) OmpA is uniquely able to couple the compressive strength of the OMP lattice and tensile strength of the CW via β-barrel interaction, a flexible linker, and CW binding, allowing the whole cell envelope to act as a mechanically strong composite.

Since OmpA is also known to contribute to OM stiffness (29), we next studied how stiffness may be related to OmpA structure and OM order. To test this, we first measured the stiffness of ∆*ompA*, *ompA^1-191^*, and *ompX-ompA^192-346^* cells, relative to the WT, by their response to osmotic force extension. As expected, this shows that ∆*ompA* cells are significantly softer than the WT. We predict that this is also the reason for increased sensitivity to β-lactams in ∆*ompA* cells (Fig. 1), where loss of OM integrity places increased burden on the CW, so reduces tolerance to CW disruption. Neither introduction of OmpX-OmpA^192-346^ nor OmpA^1-191^ detectably improves stiffness (Fig. 4*A*). This was also reflected by whole cell stiffness measurements using resistance to oscillatory osmotic shock (29), where no constructs tested could compensate for reduced mechanical integrity (*SI Appendix*, Fig. S6). This was surprising given the slight increase in resistance to β-lactams in *ompX-ompA^192-346^* cells (Fig. 2*C*), although direct mechanical measurements are a more stringent test of mechanical strength. Overall, this shows that a fully functional OmpA is required for cell envelope strength and that none of these constructs fully compensate for loss of OmpA.

## Discussion

Since the discovery of OmpA in the 1970s, the molecular mechanism for its role in virulence, OM integrity, and adhesion (28) has remained unknown. Meanwhile, our appreciation for the diversity of diderm cell envelopes has expanded, revealing widespread distribution of OM-tethered β-barrels (41). The high expression of OmpA, combined with its small β-barrel and CW attachment, make it uniquely able to act as an OM organizer and a mechanical coupler: providing a direct link between the compressive strength of the OMP lattice (42) and tensile strength of the CW (7, 43). This allows the whole cell envelope to act together as a composite, mechanically stronger than the sum of its parts (Fig. 4 *B* and *C*). Similar to reinforced concrete, where concrete withstands compression and steel bars take tension, the OMP-lattice and CW can only act as a composite when the compressive and tensile components are physically bound together: Either altered OMP-lattice (Fig. 4*A*; *ompX-ompA^192-346^*) or CW interactions (Fig. 4*A*; *ompA^1-191^*) prevent mechanical coupling, reducing the strength of the cell envelope.

The mechanical role of the OM is now well appreciated, with the balance between CW strength and OM content playing a key role (3, 29, 30). We know that a strengthened OM can compensate for cell shape defects due to weakening of the CW (30) and that the insertion of OM components is intrinsically linked to CW synthesis (44, 45). It is also known that changes to the CW affect mechanical properties (3) and simulations show that the presence of OMPs increases OM rigidity (42). The importance of the linkage itself has also been established by simulations where the cell envelope stiffness is enhanced by the presence of OMPs that are bound to the CW, whereas no such increase is found for unbound OMPs (46). Experimentally, it has been shown that the CW must be coupled by Lpp and OmpA to balance high turgor pressures (47), and OM compression can balance tensile stress in a highly stretched CW during plasmolysis (29).

Interestingly, the coupling of the CW and OM results in a strongly asymmetric material, suggesting that the mechanical response of the envelope differs when pushed outward or inward, which could impact envelope events such as blebbing or cell division. Furthermore, the β-barrel of OmpA is connected to the CW binding domain by a disordered region of linker, unlike the rigid trimeric coiled coil of the Lpp linker (48). We speculate that the OmpA linker provides some flexibility to the envelope, allowing it room to bend, which is essential for accommodating cell shape. This model of OM–CW interactions also raises the question of the precise role Lpp is playing. Since it does not appear to affect OM outer leaflet organization (Fig. 2*C*), it is perhaps minimizing vesiculation. Overall, we have shown that OmpA acts as a small, stable fencepost that sits in the OM, ordering the OMP lattice and combining its strength with the CW (Fig. 4 *B* and *C*). An ordered OMP lattice provides resistance to OM outer leaflet disruption and the composite strength allows cells to resist osmotic shock.

## Materials and Methods

### Bacterial Strains and Growth Conditions.

Strains, plasmids, primers, and other oligos are listed in *SI Appendix*, Tables S1–S4. Deletions were generated by P1 transduction (49) of keio collection alleles (50) into MG1655. For clean deletions, the kanamycin cassettes were removed using FLP recombinase with pCP20(51). The *ompA^D262A/R277A^* point mutations were made by subsequent rounds of recombineering by CspRecT (52) using oligos in *SI Appendix*, Table S4. All other *ompA* alleles were made using λ red recombineering (53). *ompA^1-191^* was generated by replacing the C-terminal domain of *ompA* with a kanamycin cassette flanked by FRT sites, moved into MG1655 with selection on kanamycin and the cassette removed using FLP recombinase (51). All remaining *ompA* alleles were made by positive-negative selection using the *tetA*-*sacB* cassette (54): *tetA-sacB* was first inserted at the *ompA* locus by positive selection for tetracycline resistance, recombineering was then repeated with the alternative *ompA* allele using negative selection for growth in the presence of sucrose. Removal of *ompA* compromises the OM, so the introduction of *sacB* was particularly toxic, even in the absence of sucrose. Therefore, the second λ red recombination to remove the *tetA-sacB* cassette was performed in LB with 0.4 % glucose. Markerless *ompA* alleles were transduced into MG1655 *pyrD*::*kan*, selected by growth on minimal media agar plates, and checked by PCR.

Fragments used for λ red recombineering were made using multiple rounds of PCR. The tetA-sacB fragment was amplified from pEXG2_tetAsacB using GB96 and GB97. The kanamycin cassette for the truncation fragment was amplified from MG1655 *lpp::kan* genomic DNA using primers GB99 and GB100. For the *ompX-ompA^192-346^* fragment, MG1655 genomic DNA was amplified using two primer pairs (GB86/GB88 and GB87/GB89) these fragments were then used for overlapping pcr (55) using GB86 and GB89, 5’ and 3’ overhangs were then added by amplification with GB90 and GB91. To improve recombination efficiency, the *ompX-ompA^192-346^* fragment overlapping regions were subsequently extended by amplification with GB106 and GB107. For the lpp^1-35^-ompA^195-346^ fragment, lpp was amplified by GB125/GB127 and ompA by GB126/GB128, these were purified and combined in an overlap PCR with GB129/GB130. For the *ompX* fragment, MG1655 genomic DNA was amplified using GB135/136. For the *ompX-ompA^192-346:D262A/R277A^* fragment, the construct was first made on a plasmid, then amplified using GB116/117.

### OM Extraction, SDS-PAGE, and Western Blotting.

Membranes were purified by urea extraction as described in ref. 56. 8 µL of samples from membrane extraction was loaded into 15% SDS-PAGE gels, run at 100 V for 1 h 30 min, transferred onto nitrocellulose membranes, and blocked in 5% milk at room temperature for 1 h. Where membrane extraction was not required, whole cell lysates were generated by resuspending 20 µL of cells at an OD600 of 50 in 20 µL lysis buffer (1 mM EDTA, 0.5 mg/mL lysozyme, and 50 mM TRIS pH 6.8), performing 4 freeze-thaws at −80 °C-room temperature, incubating with 2.5 µL 0.1 × benzonase for 15 min at room temperature, adding 20 uL of sample buffer (Laemmli and β-mercaptoethanol) and splitting samples. OM protein folding was assessed by loading SDS-PAGE gel with 8 µL sample kept at room temperature and 8 µL boiled for 10 min. OmpF/C with GroEL blots used 10% SDS-PAGE gels, run at 100 V for 1 h, all other blots were run as described above. Primary antibodies for the C-terminal domain of OmpA (1:10,000), loop 4 of OmpA (1:5,000; kindly donated by Harris Bernstein), GroEL (1:70,000), OmpX (1:2,000; Invitrogen), or OmpF/C (1:50,000) were incubated in 5% milk at 4 °C overnight. The secondary antibody was goat anti-rabbit IgG-peroxidase (1:10,000) and incubated in 5% milk for 1 h at room temperature. Due to low efficiency of the antibody, 15 µL of sample was loaded for anti-OmpX blots. All membrane purifications and westerns were performed in triplicate.

### Efficiency of Plating Assays.

Bacteria were grown in LB at 37 °C overnight. 100 µL overnight culture was inoculated into 5 mL LB and incubated at 37 °C for 1 h. The OD600 was normalized to 1, and serial 10-fold dilutions were made in a 96-well plate. Cells were then transferred using a replica plater onto LB agar containing 1% bile salts, 1 mM EDTA, 2 µg/mL ampicillin, 2 µg/mL chloramphenicol, and 25 µg/mL cefsulodin. Plates were left at 37 °C overnight except bile salt plates which were incubated at room temperature for 48 h. All EOPs were performed in triplicate.

### AFM and Image Analysis.

Bacteria and coverslips were prepared as described previously in ref. 14, using a 1.5 to 2 h exponential growth. Dynamic (AC) AFM imaging was performed using microscopes, cantilevers, and parameters described previously in ref. 14 with 500 × 500 nm scan size, 512 × 512 pixels and 5 Hz line rate. Each image type was acquired on a minimum of 3 cells per sample and each sample was performed in biological and technical triplicate.

AFM images were processed to remove noise and background cell curvature in Pygwy [using Gwyddion (57)]. Briefly, height and phase images were processed by applying a 2 nd-order polynomial line subtraction, the fix zero function, and a 1 pixel Gaussian filter. To flatten images sufficiently to find minima, imageJ (58) was used to apply a 1 to 50 pixel bandpass filter, then a 1 pixel Gaussian filter was applied, the mean zeroed, and converted to a 32-bit file. For image representation, Gwyddion was then used to set the color scales. Line profiles were taken on processed images, to avoid cell curvature dominating the profile, using gwyddion.

Since phase images showed the same lattice information as height images, only at reliably higher resolution, the phase was used throughout to study OMP lattice integrity. To quantify the quality of pores in an image, 32-bit processed files were converted to 8-bit to normalize the pixel range. An ideal pore was first generated by applying the Find Maxima function, in ImageJ (58), to identify the coordinates of pores in MG1655 wild-type images. This was approximately 1,500 pores per 500 nm image. In MATLAB (MathWorks), a 16×16 pixel square was cropped out around each pore coordinate and the resulting images averaged (approximately 20,500 pores were averaged) to find the ideal WT pore. To quantify lattice quality, test pore coordinates were found for each image by applying the Find Maxima function in ImageJ, increasing the threshold by five until the number of maxima was below the expected number of pores (1,500 pores per 500 nm image). This was to prevent oversampling of noisy, low-resolution images with many small minima. MATLAB (MathWorks) was then used similarly to above, where a box was cropped around each coordinate, but this time, the normalized 2D cross correlation function was applied to compare the ideal pore with the test pore, and the highest correlation coefficient of the crop found. To get a quantification where the higher the number, the lower resolution, the peak correlation was subtracted from 1. This was done for all coordinates in an image and the mean taken to represent the lattice quality of the image. This process is summarized in *SI Appendix*, Fig. S4. Pygwy, ImageJ, and MATLAB scripts used can be found in the appendix.

### Mass Spectrometry.

Samples were prepared as previously described (59). Briefly, each sample containing ~250 µg of total protein was lyophilized to remove water and then resuspended in 250 µL of lysis buffer containing 50 mM HEPES pH 7.2, 2% CTAB (hexadecyltrimethylammonium bromide), 6 M GuHCl (guanidine hydrochloride), and 5 mM DTT. Cells were lysed by direct tip sonication for 10 cycles of 30 s each at 50% amplitude and further heating the lysate at 60 °C for 20 min. Lysate was then methanol-chloroform precipitated to extract proteins. Proteins were digested in a two-step LysC (Wako) + LysC/Trypsin (Promega, sequencing grade) protocol in 10 mM EPPS pH 8.5 with 2/0.5 M GuCl. The first digestion was performed at room temperature and the second at 37 °C. The digested samples were dried using a vacuum evaporator at room temperature and taken up in 200 mM EPPS pH 8.0. The multiplexing TMTpro tags were added at a mass ratio of 5:1 tag/peptide to ~10 µg of peptide per condition and allowed to react for 2 h at room temperature before quenching with 1% hydroxylamine (30 min, room temperature). Samples from all conditions were combined into one tube and acidified with 5% phosphoric acid (pH <2). The samples were then ultracentrifuged at 100,000×*g* at 4 °C for an hour to pellet undigested proteins. The supernatants were dried using a vacuum evaporator at room temperature to remove acetonitrile. Dry samples were resuspended in HPLC water and stage-tipping was performed to desalt the samples (60).

For LC–MS analysis, samples were resuspended to 1 µg/µL in 1% FA and HPLC-grade water. Samples were analyzed on a nLC-1200 HPLC coupled to an Orbitrap Fusion Lumos mass spectrometer (Thermo Fisher Scientific). Peptides were separated on an Aurora Series emitter column (25 cm x 75 µm ID, 1.6 µm C18, Ionopticks), held at 60 °C during separation by an in-house built column oven. Samples were eluted with a 12 to 24% acetonitrile gradient over 90 min. Peptide quantification was done with TMTproC using the Gygi Lab GFY software licensed from Harvard (61). Briefly, the complement reporter ion cluster was located, and the observed intensities were extracted. Using the measured shape of the isolation window and measured TMTpro isotopic impurities, the relative abundance and composition of each isolated peak was determined and used in the deconvolution algorithm. Peptides with a total signal:Fourier transform noise less than 30 were discarded, and proteins with less than two peptides were discarded. Estimates of protein fold-change were calculated from the median ratios of peptides assigned uniquely to that protein sequence. Statistical significance was determined by performing a *t* test without multiple hypothesis correction on triplicate measurements within the same plex.

### Microfluidics Stiffness Measurements.

Cells were imaged on a Nikon Eclipse Ti-E inverted fluorescence microscope with a 100× (NA 1.45) oil-immersion objective in CellASIC B04A microfluidic perfusion plates and medium was exchanged using the CellASIC ONIX2 microfluidic platform. Images were collected on a DU885 electron-multiplying charge-coupled device camera (Andor) using μManager v.1.426. Cells were maintained at 37 °C during imaging with an active-control environmental chamber (HaisonTech).

Oscillatory osmotic shocks were used to calculate whole cell stiffness, as described previously (29). Briefly, overnight cultures were diluted 100-fold into 1 mL of fresh LB and incubated for 2 h with shaking at 37 °C. Plates were loaded with medium prewarmed to 37 °C. Cells were then loaded into the plate and incubated at 37 °C, without shaking, for 30 min before imaging. Cells were then allowed to grow for 5 min in LB medium while imaging in the chamber before being subjected to 600-mM oscillatory osmotic shocks by switching between LB and LB with 600 mM sorbitol. To measure lysis curves, a cell was considered to have lysed when its cell size ceased to oscillate in response to the osmotic shocks.

Osmotic force extension was used to calculate relative cell envelope stiffness as described previously (62). Overnight cultures were diluted 100-fold into 1 ml of fresh LB and incubated for 2.5 h with shaking at 37 °C. Plates were loaded with medium prewarmed to 37 °C. Cells were loaded into the plate at 37 °C, directly before imaging. Multiple strains were measured simultaneously by loading strains dyed before loading into the same chamber and identifying each strain in postprocessing. The cell envelope was stained with the fluorescent D-amino acid HADA (Tocris Bioscience; 250 µM), MitoTracker Orange CM-H2TMRos (Invitrogen; 500 nM), MitoView Green (Biotium; 200 nM), or MitoView 720 (Biotium; 100 nM). To be sure that measurements were not affected by dyes, different dyes were used in each strain for each repeat. Cells were then grown while imaging for 5 min in LB before being serially plasmolyzed using LB with 50, 100, 200, and 400 mM sorbitol for 1 min each. Between sorbitol shocks, the medium was switched to LB for 1 min.

To calculate the amplitude of length oscillations during osmotic shocks, cells were tracked using custom MATLAB algorithms. First, cell-envelope lengths (*l*) were automatically detected and the elongation rate (*e* = *d*(ln*l*)/*dt*) was calculated for each cell. The effective population-averaged length was calculated by integrating the population-averaged elongation rate over time (63). The mechanical strain (ε = *l1- l2*/*l2*) in cell-envelope length was calculated for each shock. Linear regression of mechanical strain as a function of shock magnitude was calculated where cell-envelope stiffness was defined as 1/slope. Uncertainty was estimated using the SE of the linear regression model’s slope.

### Simulations.

The structures of OmpF, FhuA, and OmpA were obtained from X-ray crystallography data PDB:3K1B (64), PDB:1FCP (65), and PDB:1BXW (66), respectively. These structures were coarse-grained with default settings using the martinize.py script and the Martini 2.2 forcefield (15). The insane.py script (67) was used to build the coarse-grained membrane model and insert the proteins in them. The membrane used 100% fast ReLPS (38) in the outer leaflet and a 90/5/5 ratio of 16:0 to 18:1 phosphoethanolamine (POPE)/16:0 to 18:1 palmitoylphosphatidylglycerol (POPG)/cardiolipin in the inner leaflet. After inserting the proteins in the membrane (29 copies of OmpF trimers, 17 copies of FhuA, and 44 copies of OmpA), the simulation system was solvated with 0.15 M Na^+^ and Cl^−^ ions and further neutralized with Na^+^ ions. The fully solvated system was then energy-minimized using the steepest descent algorithm until forces converged to less than 1,000 kJ/mol/nm. Then, it was subjected to a total equilibration time of 1,100 ns using a 20 fs timestep. The end of the equilibration simulations were then used to generate seeds for three individual 2.75 µs-long production simulations with differing initial velocities. The simulation box dimensions along the membrane plane measured 64.7 × 68.5 nm^2^ after equilibrating it in the presence of Martini water, 0.15 M NaCl, and neutralizing ions. Both equilibration and production simulations were conducted at a temperature of 340 K maintained by a velocity rescaling thermostat (68). A high temperature was maintained to provide more sampling during the simulation timescale. The pressure was maintained at 1 bar with semi-isotropic pressure coupling using the Berendsen barostat (69) during equilibration and using the Parrinello Rahman barostat (70) during production. The van der Waals and coulombic radii were both set to 1.1 nm and treated with the cutoff algorithm along with the potential-shift modifier. All simulations were performed using the Gromacs simulation package (71). The MSD and density analyses were performed using the gmx msd and gmx densmap tools of Gromacs, respectively. The diffusion coefficient was calculated using the equation: D = MSD slope/4dt, where “4” is a constant since the MSD was calculated only along the plane of the membrane, and “dt” is the time difference (500 ns) within which the MSD curve was used to calculate a slope. The MSD slope calculated here was a linear regression of the MSD curve between 2,000 to 2,500 ns. The slopes for the OmpA-NR and OmpA-R simulations were 0.0009276884 and 0.0001171221, respectively. Visual Molecular Dynamics (72) was used for visualization and making figures. Matplotlib (73) was used to plot the data. Density maps of all three simulation repeats are shown in *SI Appendix*, Fig. S7.

### Statistics, Data Presentation, and Bioinformatics.

Two-sided paired *t* tests were performed in MATLAB (MathWorks). Graphing and line profiles were visualized in Origin Pro (OriginLab), including finding means, SD, and OM mechanics one-sided *t* tests. Figures were made using Pymol (74) and Adobe Illustrator. Amino acid sequence similarity was assessed by comparison of P0A910 (OmpA) with P0A917 (OmpX) in BLASTp.

## Supplementary Material

Appendix 01 (PDF)

## Data Availability

The mass spectrometry proteomics data have been deposited to the ProteomeXchange Consortium via the PRIDE partner repository with the dataset identifier PXD054033 (75). Other replicates and data can be found at Princeton Data Commons (76) (https://doi.org/10.34770/ymvr-mg79).

## References

[1] C. J. Murray , Global burden of bacterial antimicrobial resistance in 2019: A systematic analysis. Lancet **399**, 629–655 (2022). 35065702 10.1016/S0140-6736(21)02724-0 PMC8841637

[2] D. J. Payne, M. N. Gwynn, D. J. Holmes, D. L. Pompliano, Drugs for bad bugs: Confronting the challenges of antibacterial discovery. Nat. Rev. Drug Discov. **6**, 29–40 (2007). 17159923 10.1038/nrd2201

[3] J. Sun, S. T. Rutherford, T. J. Silhavy, K. Casey Huang, Physical properties of the bacterial outer membrane. Nat. Rev. Microbiol. **20**, 236–248 (2022). 34732874 10.1038/s41579-021-00638-0 PMC8934262

[4] S. I. Miller, N. R. Salama, The gram-negative bacterial periplasm: Size matters. PLoS Biol. **16**, e2004935 (2018). 29342145 10.1371/journal.pbio.2004935 PMC5771553

[5] T. J. Silhavy, D. Kahne, S. Walker, The bacterial cell envelope. Cold Spring Harb. Perspect Biol. **2**, a000414 (2010), 10.1101/cshperspect.a000414. 20452953 PMC2857177

[6] E. Hoiczyk, W. Baumeister, Envelope structure of four gliding filamentous cyanobacteria. J. Bacteriol. **177**, 2387–2395 (1995). 7730269 10.1128/jb.177.9.2387-2395.1995 PMC176896

[7] J.-V. Höltje, Growth of the stress-bearing and shape-maintaining murein sacculus of Escherichia coli. Microbiol. Mol. Biol. Rev. **62**, 181–203 (1998). 9529891 10.1128/mmbr.62.1.181-203.1998 PMC98910

[8] W. Vollmer, D. Blanot, M. A. De Pedro, Peptidoglycan structure and architecture. FEMS Microbiol. Rev. **32**, 149–167 (2008). 18194336 10.1111/j.1574-6976.2007.00094.x

[9] L. Gan, S. Chen, G. J. Jensen, Molecular organization of gram-negative peptidoglycan. Proc. Natl. Acad. Sci. **105**, 18953–18957 (2008). 19033194 10.1073/pnas.0808035105 PMC2596242

[10] J. Witwinowski , An ancient divide in outer membrane tethering systems in bacteria suggests a mechanism for the diderm-to-monoderm transition. Nat. Microbiol. **7**, 411–422 (2022). 35246664 10.1038/s41564-022-01066-3

[11] D. Jefferies, J. Shearer, S. Khalid, Role of O-antigen in response to mechanical stress of the *E. coli* outer membrane: Insights from coarse-grained MD simulations. J. Phys. Chem. B **123**, 3567–3575 (2019). 30971088 10.1021/acs.jpcb.8b12168

[12] H. Nikaido, Molecular basis of bacterial outer membrane permeability revisited. Microbiol. Mol. Biol. Rev. **67**, 593–656 (2003). 14665678 10.1128/MMBR.67.4.593-656.2003 PMC309051

[13] G.-W. Li, D. Burkhardt, C. Gross, J. S. Weissman, Quantifying absolute protein synthesis rates reveals principles underlying allocation of cellular resources. Cell **157**, 624–635 (2014). 24766808 10.1016/j.cell.2014.02.033 PMC4006352

[14] G. Benn , Phase separation in the outer membrane of Escherichia coli. Proc. Natl. Acad. Sci. **118**, e2112237118 (2021). 34716276 10.1073/pnas.2112237118 PMC8612244

[15] M. N. Webby , Lipids mediate supramolecular outer membrane protein assembly in bacteria. Sci. Adv. **8**, eadc9566 (2022). 36322653 10.1126/sciadv.adc9566 PMC9629720

[16] D. L. Sexton , The cell envelope of Thermotogae suggests a mechanism for outer membrane biogenesis. Proc. Natl. Acad. Sci. **120**, e2303275120 (2023). 37094164 10.1073/pnas.2303275120 PMC10160955

[17] M. E. Bayer, C. Remsen, Structure of *Escherichia coli* after freeze-etching. J. Bacteriol. **101**, 304–313 (1970). 4189229 10.1128/jb.101.1.304-313.1970 PMC250482

[18] Z. Oestreicher, A. Taoka, Y. Fukumori, A comparison of the surface nanostructure from two different types of gram-negative cells: *Escherichia coli* and *Rhodobacter sphaeroides*. Micron **72**, 8–14 (2015). 25725215 10.1016/j.micron.2015.02.001

[19] S. Jaroslawski, K. Duquesne, J. N. Sturgis, S. Scheuring, High-resolution architecture of the outer membrane of the Gram-negative bacteria Roseobacter denitrificans. Mol. Microbiol. **74**, 1211–1222 (2009). 19843216 10.1111/j.1365-2958.2009.06926.x

[20] L. Thomas , Surface topography and molecular stoichiometry of the mitochondrial channel, VDAC, in crystalline arrays. J. Struct. Biol. **106**, 161–171 (1991). 1725124 10.1016/1047-8477(91)90085-b

[21] B. W. Hoogenboom, K. Suda, A. Engel, D. Fotiadis, The supramolecular assemblies of voltage-dependent anion channels in the native membrane. J. Mol. Biol. **370**, 246–255 (2007). 17524423 10.1016/j.jmb.2007.04.073

[22] R. L. Guest, T. J. Silhavy, Cracking outer membrane biogenesis. Biochim. Biophys. Acta BBA - Mol. Cell Res. **1870**, 119405 (2023). 10.1016/j.bbamcr.2022.119405 PMC9878550 36455781

[23] J. Szczepaniak , The lipoprotein Pal stabilises the bacterial outer membrane during constriction by a mobilisation-and-capture mechanism. Nat. Commun. **11**, 112–114 (2020). 32161270 10.1038/s41467-020-15083-5 PMC7066135

[24] M. Mathelié-Guinlet, A. T. Asmar, J.-F. Collet, Y. F. Dufrêne, Lipoprotein Lpp regulates the mechanical properties of the *E. coli* cell envelope. Nat. Commun. **11**, 1789 (2020). 32286264 10.1038/s41467-020-15489-1 PMC7156740

[25] M. L. Ortiz-Suarez, F. Samsudin, T. J. Piggot, P. J. Bond, S. Khalid, Full-length OmpA: Structure, function, and membrane interactions predicted by molecular dynamics simulations. Biophys. J. **111**, 1692–1702 (2016). 27760356 10.1016/j.bpj.2016.09.009 PMC5071624

[26] J. P. Rosenbusch, Characterization of the major envelope protein from *Escherichia coli*. J. Biol. Chem. **249**, 8010–8029 (1974). 4609976

[27] U. Henning, W. Schmidmayr, I. Hindennach, Major proteins of the outer cell envelope membrane of Escherichia coli K-12: Multiple species of protein I. Mol. Gen. Genet. MGG **154**, 293–298 (1977). 337109 10.1007/BF00571285

[28] S. G. J. Smith, V. Mahon, M. A. Lambert, R. P. Fagan, A molecular Swiss army knife: OmpA structure, function and expression. FEMS Microbiol. Lett. **273**, 1–11 (2007). 17559395 10.1111/j.1574-6968.2007.00778.x

[29] E. R. Rojas , The outer membrane is an essential load-bearing element in Gram-negative bacteria. Nature **559**, 617–621 (2018). 30022160 10.1038/s41586-018-0344-3 PMC6089221

[30] E. M. Fivenson , A role for the Gram-negative outer membrane in bacterial shape determination. Proc. Natl. Acad. Sci. **120**, e2301987120 (2023). 37607228 10.1073/pnas.2301987120 PMC10469335

[31] I. Sonntag, H. Schwarz, Y. Hirota, U. Henning, Cell envelope and shape of *Escherichia coli*: Multiple mutants missing the outer membrane lipoprotein and other major outer membrane proteins. J. Bacteriol. **136**, 280–285 (1978). 361695 10.1128/jb.136.1.280-285.1978 PMC218658

[32] C. Kleanthous, P. Rassam, C. G. Baumann, Protein-protein interactions and the spatiotemporal dynamics of bacterial outer membrane proteins. Curr. Opin. Struct. Biol. **35**, 109–115 (2015). 26629934 10.1016/j.sbi.2015.10.007 PMC4684144

[33] M. Grabowicz, T. J. Silhavy, Envelope stress responses: An interconnected safety net. Trends Biochem. Sci. **42**, 232–242 (2017). 27839654 10.1016/j.tibs.2016.10.002 PMC5336467

[34] K. Dekoninck , Defining the function of OmpA in the Rcs stress response. eLife **9**, e60861 (2020). 32985973 10.7554/eLife.60861 PMC7553776

[35] J. Shearer, D. Jefferies, S. Khalid, Outer membrane proteins OmpA, FhuA, OmpF, EstA, BtuB, and OmpX have unique lipopolysaccharide fingerprints. J. Chem. Theory Comput. **15**, 2608–2619 (2019). 30848905 10.1021/acs.jctc.8b01059

[36] B. W. Hoogenboom, Stretching the resolution limit of atomic force microscopy. Nat. Struct. Mol. Biol. **28**, 629–630 (2021). 34294922 10.1038/s41594-021-00638-x

[37] S. J. Marrink, H. J. Risselada, S. Yefimov, D. P. Tieleman, A. H. de Vries, The MARTINI force field: Coarse grained model for biomolecular simulations. J. Phys. Chem. B **111**, 7812–7824 (2007). 17569554 10.1021/jp071097f

[38] A. F. Brandner, D. Prakaash, A. Blanco González, F. Waterhouse, S. Khalid, Faster but not sweeter: A model of Escherichia coli re-level lipopolysaccharide for Martini 3 and a Martini 2 version with accelerated kinetics. J. Chem. Theory Comput. **20**, 6890–6903 (2024), 10.1021/acs.jctc.4c00374. 39008538 PMC11325540

[39] F. Samsudin, M. L. Ortiz-Suarez, T. J. Piggot, P. J. Bond, S. Khalid, OmpA: A flexible clamp for bacterial cell wall attachment. Structure **24**, 2227–2235 (2016). 27866852 10.1016/j.str.2016.10.009

[40] J. S. Park , Mechanism of anchoring of OmpA protein to the cell wall peptidoglycan of the gram-negative bacterial outer membrane. FASEB J. **26**, 219–228 (2012). 21965596 10.1096/fj.11-188425 PMC3250236

[41] A. Silale , Dual function of OmpM as outer membrane tether and nutrient uptake channel in diderm Firmicutes. Nat. Commun. **14**, 7152 (2023). 37932269 10.1038/s41467-023-42601-y PMC10628300

[42] H. J. Lessen, P. J. Fleming, K. G. Fleming, A. J. Sodt, Building blocks of the outer membrane: Calculating a general elastic energy model for β-barrel membrane proteins. J. Chem. Theory Comput. **14**, 4487–4497 (2018). 29979594 10.1021/acs.jctc.8b00377 PMC6191857

[43] Y. Deng, M. Sun, J. W. Shaevitz, Direct measurement of cell wall stress stiffening and turgor pressure in live bacterial cells. Phys. Rev. Lett. **107**, 158101 (2011). 22107320 10.1103/PhysRevLett.107.158101

[44] K. R. Hummels , Coordination of bacterial cell wall and outer membrane biosynthesis. Nature **615**, 300–304 (2023). 36859542 10.1038/s41586-023-05750-0 PMC9995270

[45] G. Mamou , Peptidoglycan maturation controls outer membrane protein assembly. Nature **606**, 953–959 (2022). 35705811 10.1038/s41586-022-04834-7 PMC9242858

[46] D. Ryoo, H. Hwang, J. C. Gumbart, Thicket and mesh: How the outer membrane can resist tension imposed by the cell wall. J. Phys. Chem. B **128**, 5371-5377 (2024), 10.1021/acs.jpcb.3c08510. 38787347 PMC11163421

[47] M. Deghelt , The outer membrane and peptidoglycan layer form a single mechanical device balancing turgor. bioRxiv [Preprint], (2023), 10.1101/2023.04.29.538579 (Accessed 2 July 2024).

[48] A. T. Asmar, J.-F. Collet, Lpp, the Braun lipoprotein, turns 50—major achievements and remaining issues. FEMS Microbiol. Lett. **365**, fny199 (2018). 10.1093/femsle/fny199 30107563

[49] T. Silhavy, M. Berman, L. Enquist, Experiments with Gene Fusions (Cold Spring Harb. Lab., NY, 1984).

[50] T. Baba , Construction of *Escherichia coli* K-12 in-frame, single-gene knockout mutants: The Keio collection. Mol. Syst. Biol. **2**, 2006.0008 (2006). 10.1038/msb4100050 PMC1681482 16738554

[51] P. P. Cherepanov, W. Wackernagel, Gene disruption in *Escherichia coli*: TcR and KmR cassettes with the option of Flp-catalyzed excision of the antibiotic-resistance determinant. Gene **158**, 9–14 (1995). 7789817 10.1016/0378-1119(95)00193-a

[52] T. M. Wannier , Improved bacterial recombineering by parallelized protein discovery. Proc. Natl. Acad. Sci. **117**, 13689–13698 (2020). 32467157 10.1073/pnas.2001588117 PMC7306799

[53] L. C. Thomason, N. Costantino, X. Li, D. L. Court, Recombineering: Genetic engineering in *Escherichia coli* using homologous recombination. Curr. Protoc. **3**, e656 (2023). 36779782 10.1002/cpz1.656 PMC10037674

[54] X. Li, L. C. Thomason, J. A. Sawitzke, N. Costantino, D. L. Court, Positive and negative selection using the tetA-sacB cassette: Recombineering and P1 transduction in *Escherichia coli*. Nucleic Acids Res. **41**, e204 (2013). 24203710 10.1093/nar/gkt1075 PMC3905872

[55] R. S. Hilgarth, T. M. Lanigan, Optimization of overlap extension PCR for efficient transgene construction. MethodsX **7**, 100759 (2020). 32021819 10.1016/j.mex.2019.12.001 PMC6992990

[56] A. N. Combs, T. J. Silhavy, The sacrificial adaptor protein Skp functions to remove stalled substrates from the β-barrel assembly machine. Proc. Natl. Acad. Sci. **119**, e2114997119 (2022). 34969846 10.1073/pnas.2114997119 PMC8740687

[57] D. Nečas, P. Klapetek, Gwyddion: An open-source software for SPM data analysis. Cent. Eur. J. Phys. **10**, 181–188 (2012).

[58] J. Schindelin , Fiji: An open-source platform for biological-image analysis. Nat. Methods **9**, 676–682 (2012). 22743772 10.1038/nmeth.2019 PMC3855844

[59] M. Gupta , Global protein turnover quantification in *Escherichia coli* reveals cytoplasmic recycling under nitrogen limitation. Nat. Commun. **15**, 5890 (2024). 39003262 10.1038/s41467-024-49920-8 PMC11246515

[60] J. Rappsilber, M. Mann, Y. Ishihama, Protocol for micro-purification, enrichment, pre-fractionation and storage of peptides for proteomics using StageTips. Nat. Protoc. **2**, 1896–1906 (2007). 17703201 10.1038/nprot.2007.261

[61] A. Johnson, M. Stadlmeier, M. Wühr, TMTpro complementary ion quantification increases plexing and sensitivity for accurate multiplexed proteomics at the MS2 level. J. Proteome Res. **20**, 3043–3052 (2021). 33929851 10.1021/acs.jproteome.0c00813 PMC8330405

[62] D. Fitzmaurice β-barrel proteins dictate the effect of core oligosaccharide composition on outer membrane mechanics. bioRxiv [Preprint] (2024). 10.1101/2024.09.02.610904 (Accessed 25 September 2024). PMC11897544 39863924

[63] E. Rojas, J. A. Theriot, K. C. Huang, Response of *Escherichia coli* growth rate to osmotic shock. Proc. Natl. Acad. Sci. **111**, 7807–7813 (2014). 24821776 10.1073/pnas.1402591111 PMC4040581

[64] G. Kefala , Structures of the OmpF porin crystallized in the presence of foscholine-12. Protein Sci. Publ. Protein Soc. **19**, 1117–1125 (2010). 10.1002/pro.369 PMC2868254 20196071

[65] A. D. Ferguson, E. Hofmann, J. W. Coulton, K. Diederichs, W. Welte, Siderophore-mediated iron transport: Crystal structure of FhuA with bound lipopolysaccharide. Science **282**, 2215–2220 (1998). 9856937 10.1126/science.282.5397.2215

[66] A. Pautsch, G. E. Schulz, Structure of the outer membrane protein A transmembrane domain. Nat. Struct. Biol. **5**, 1013–1017 (1998). 9808047 10.1038/2983

[67] T. A. Wassenaar, H. I. Ingólfsson, R. A. Böckmann, D. P. Tieleman, S. J. Marrink, Computational lipidomics with insane: A versatile tool for generating custom membranes for molecular simulations. J. Chem. Theory Comput. **11**, 2144–2157 (2015). 26574417 10.1021/acs.jctc.5b00209

[68] G. Bussi, D. Donadio, M. Parrinello, Canonical sampling through velocity rescaling. J. Chem. Phys. **126**, 014101 (2007). 17212484 10.1063/1.2408420

[69] H. J. C. Berendsen, J. P. M. Postma, W. F. van Gunsteren, A. DiNola, J. R. Haak, Molecular dynamics with coupling to an external bath. J. Chem. Phys. **81**, 3684–3690 (1984).

[70] M. Parrinello, A. Rahman, Polymorphic transitions in single crystals: A new molecular dynamics method. J. Appl. Phys. **52**, 7182–7190 (1981).

[71] M. J. Abraham , GROMACS: High performance molecular simulations through multi-level parallelism from laptops to supercomputers. SoftwareX **1–2**, 19–25 (2015).

[72] W. Humphrey, A. Dalke, K. Schulten, VMD: Visual molecular dynamics. J. Mol. Graph. **14**, 33–38 (1996). 8744570 10.1016/0263-7855(96)00018-5

[73] J. D. Hunter, Matplotlib: A 2D graphics environment. Comput. Sci. Eng. **9**, 90–95 (2007).

[74] L. L. C. Schrödinger, The PyMOL Molecular Graphics System, version **1**, 8 (2015).

[75] A. Johnson, M. Wühr, OmpA controls order in the outer membrane and shares the mechanical load. ProteomeXchange Consortium. https://proteomecentral.proteomexchange.org/cgi/GetDataset?ID=PXD054033. Deposited 17 July 2024. 10.1073/pnas.2416426121 PMC11648852 39630873

[76] G. Benn, C. Borrelli, D. Prakaash, V. A. Fideli, Raw data for “OmpA controls orderin the outer membrane and shares the mechanical load”. Princeton DataCommons. 10.34770/ymvr-mg79. Deposited 21 November 2024. PMC11648852 39630873

