# Supplementary material for: OmpA controls order in the outer membrane and shares the mechanical load
Source: Proc Natl Acad Sci U S A. 2024 Dec 4;121(50):e2416426121. doi: 10.1073/pnas.2416426121 (PMC11648852; doi:10.1073/pnas.2416426121)
Supplement: Supplementary file 1 — Appendix 01 (PDF) [file pnas.2416426121.sapp.pdf]

PNAS

Supporting Information for
OmpA controls order in the outer membrane and shares the mechanical load.

Georgina Benn, Carolina Borrelli, Dheeraj Prakaash, Alex N T Johnson, Vincent A Fideli, Tahj Starr, Dylan Fitzmaurice, Ashton N. Combs, Martin Wühr, Enrique R Rojas, Syma Khalid, Bart W. Hoogenboom, Thomas J. Silhavy

Georgina Benn
Email: georgina.benn@bioch.ox.ac.uk

This PDF file includes:

Figures S1 to S7
Tables S1 to S4
SI References

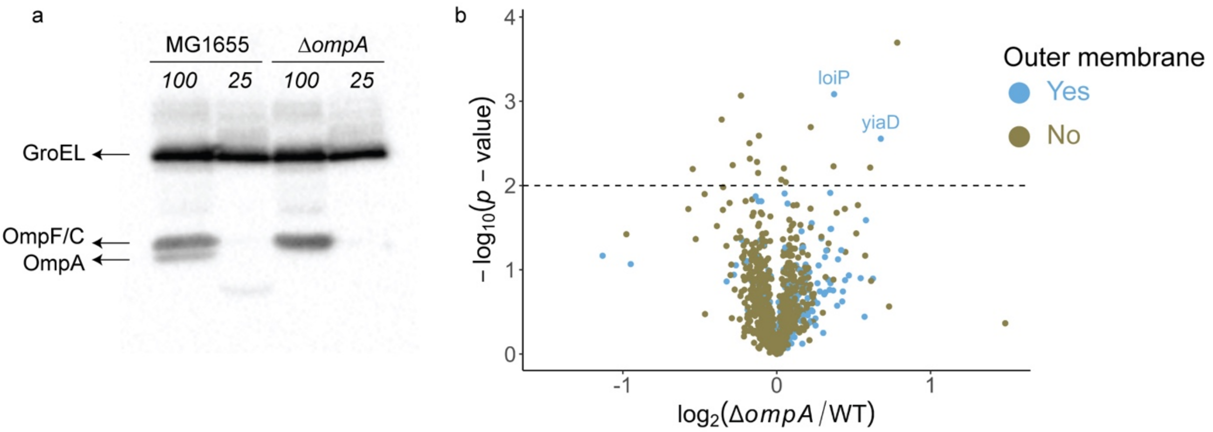

Fig. S1. OMP expression does not change when OmpA is removed. (a) Western blot for GroEL and OmpF/C of boiled (100) and unboiled (25) whole cell lysates show expression of OmpF and OmpC are unchanged by deletion of o*mpA*. OmpF/C antibody also recognises OmpA but does not recognise the folded proteins. **(b)** Quantitative mass spectrometry of whole cell lysates compared protein expression of MG1655 WT cells and Δ*ompA* cells. Points are coloured by localisation to the OM according to UniProt annotations[1]. Only the expression of the lipoproteins *loiP* and *yiaD* are increased when OmpA is removed and no OMPs are significantly altered.

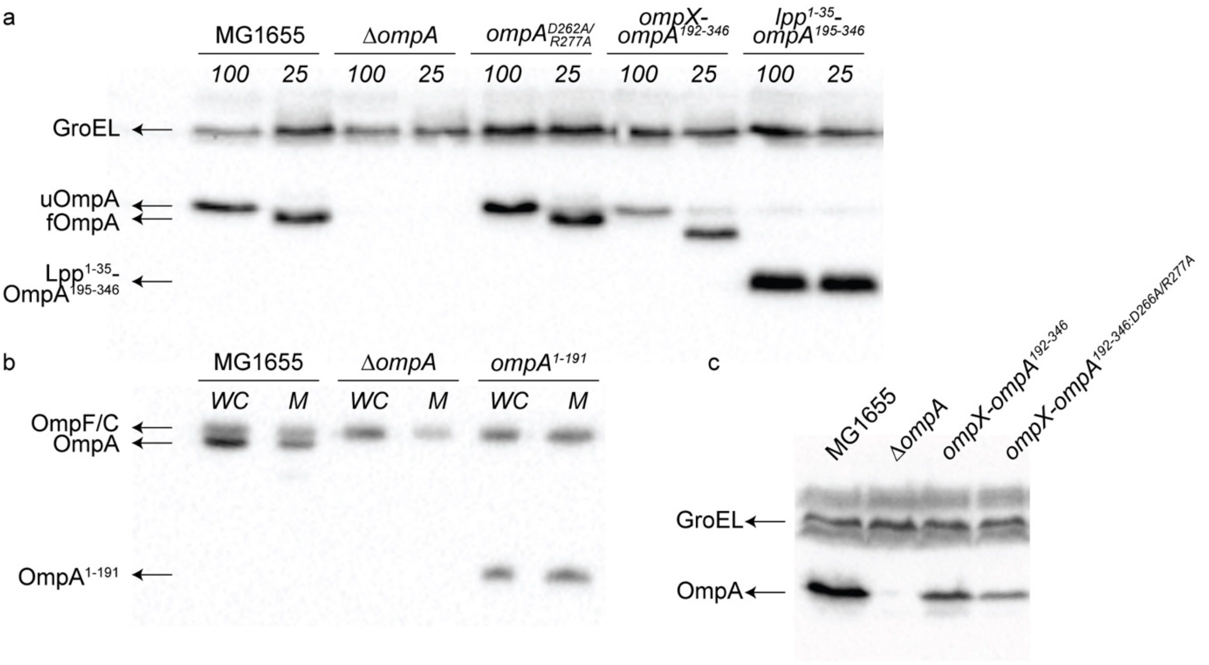

Fig. S2. Westerns blots of OmpA constructs show expression similar to wild type OmpA. All westerns were performed three independent times. **(a)** Blot for OmpA C-terminal domain and GroEL of boiled (100) and unboiled (25) whole cell lysates. OMPs are highly stable when properly folded, so run faster before boiling. Lipoproteins are not, so run at the same size with or without boiling. **(b)** Blot for OmpA Loop 4 and OmpF/C of whole cell (WC) and membrane extractions (M) show $OmpA^{1-191}$ is expressed and folded in the OM. **(c)** Blot for OmpA C-terminal domain and GroEL of MG1655, Δ*ompA,* Δ*ompA::ompX-ompA*$^{192-346}$ and Δ*ompA::ompX-ompA*$^{192-346:D262A/R277A}$ strains shows successful expression of constructs.

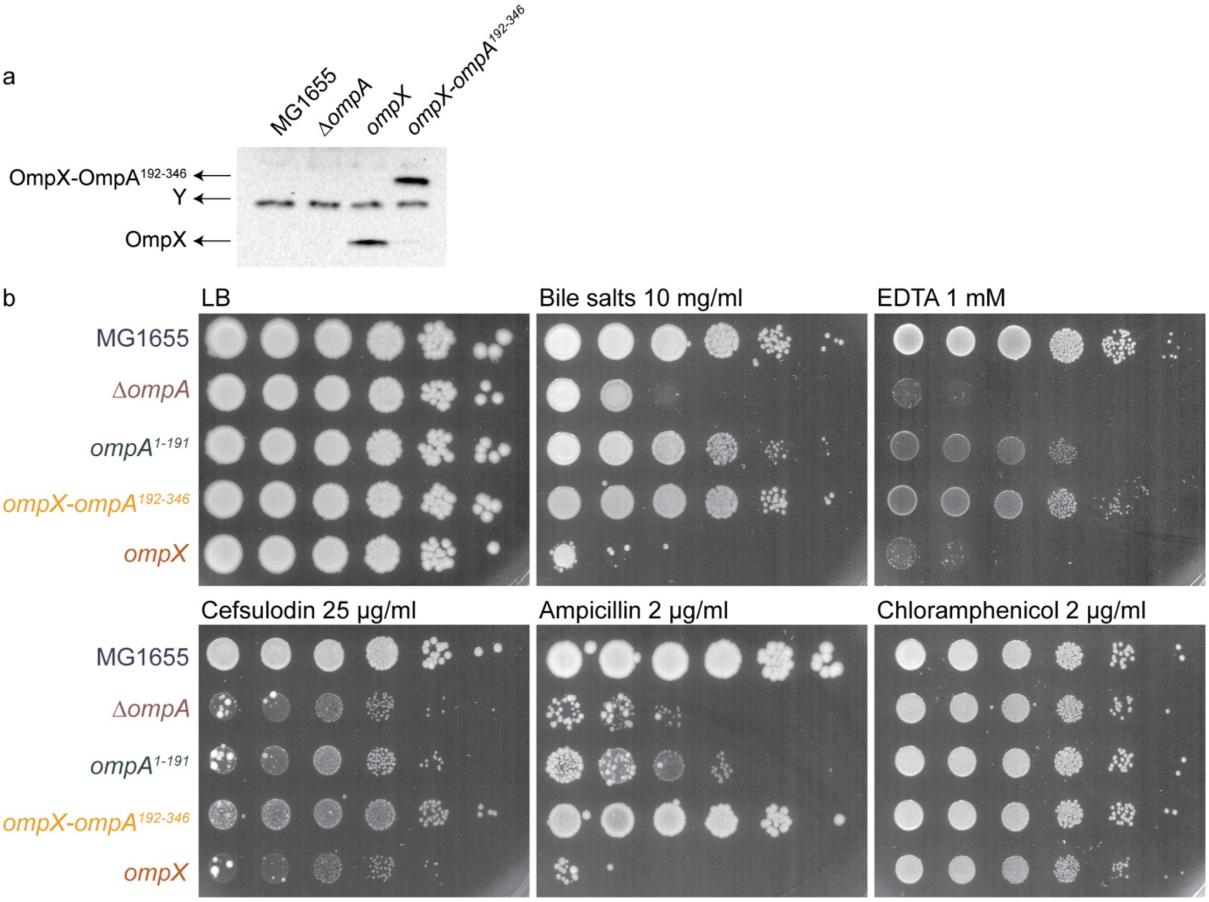

Fig. S3. Introduction of *ompX* at the *ompA* locus does not restore OM impermeability. (a) Anti-OmpX western blot of MG1655, Δ*ompA,* Δ*ompA::ompX,* Δ*ompA::ompX-ompA*$^{192\text{-}346}$ strains shows successful overexpression of OmpX. In MG1655, *ompX* expression is so low that none is detected. Non-specific band, Y, is used as loading control**. (b)** Efficiency of plating assays show introduction of *ompX* to the *ompA* locus does not improve sensitivity to any conditions tested, similar to *ompX-ompA*$^{192\text{-}346:D262A/R277A}$ (shown in Fig. 2). Dilutions from left to right are 10^0–10^{-5}.

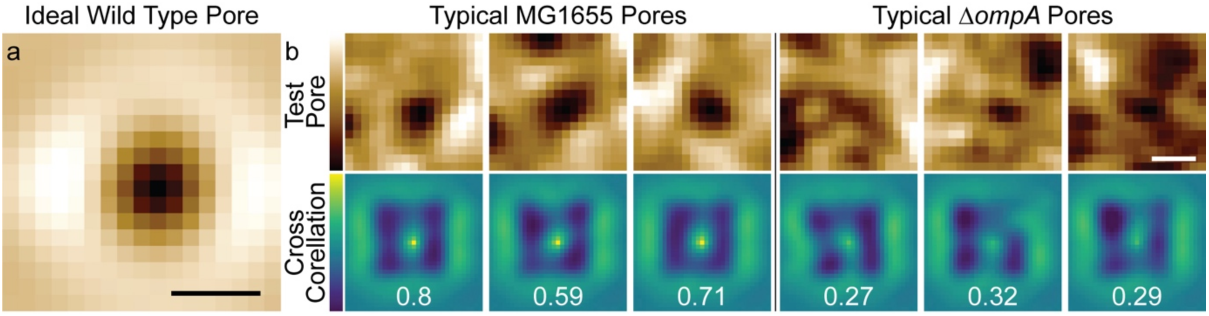

Fig. S4. Examples of AFM image analysis for lattice disruption. (a) The ideal pore for cross correlation generated by averaging all potential pores (~20,500) of all wild type images. **(b)** Single potential pores from wild type and Δ*ompA* images and, below, their cross correlation with the ideal pore. The maximum pixel value of the cross correlation is also shown. The higher the resolution of pores, the higher the peak cross correlation. To convert to lattice disruption, where the higher the number the higher the disruption, the peak cross correlation is subtracted from 1. Scale bars are 5 nm. Colour phase scale is the maximum pixel range for test pores and -0.45 to 0.58 for cross correlation.

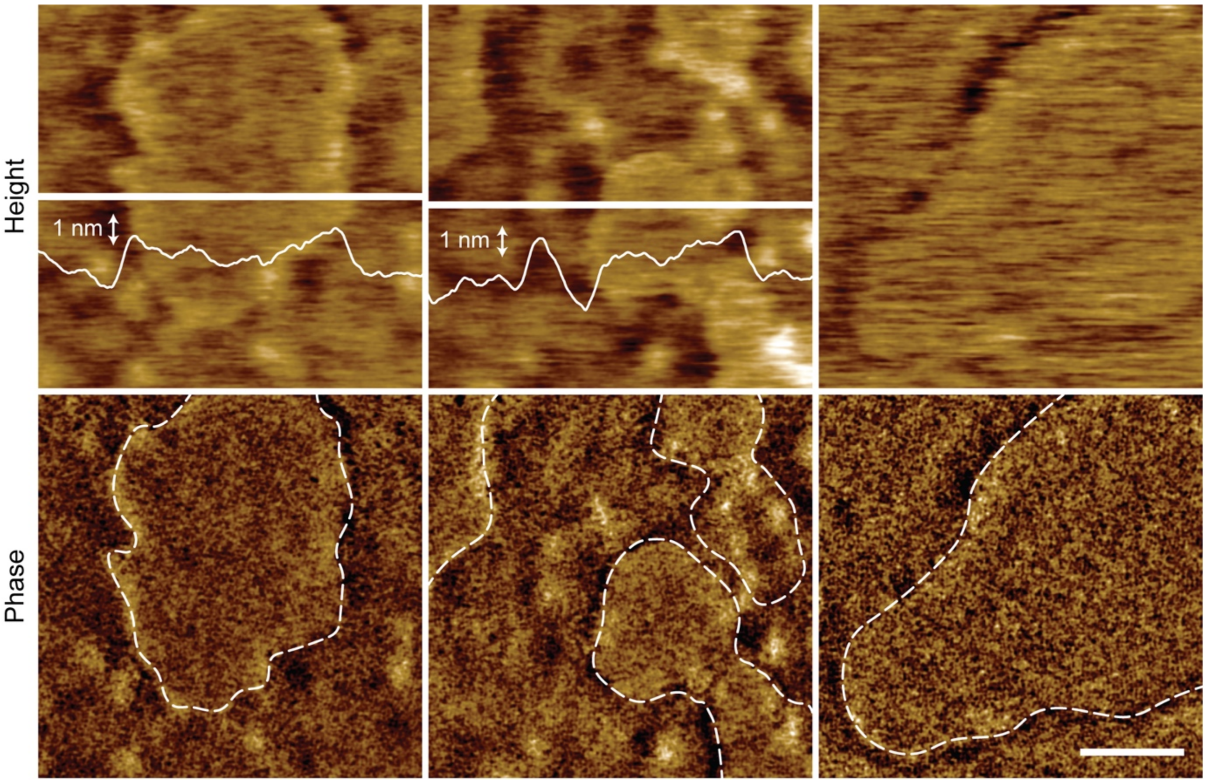

Fig. S5. AFM height and phase images of ∆*ompA* cells show large, flat plateaus 1-2 nm above the rest of the membrane. Thick solid line is location of line profile below (thin white line). White dashed line outlines approximate edges of patches, but the precise shape and size is difficult to conclusively distinguish, preventing proper quantification. Scale bar is 100 nm. Colour height scale is 5 nm and colour phase scales are 1.1, 0.95 and 0.5 deg.

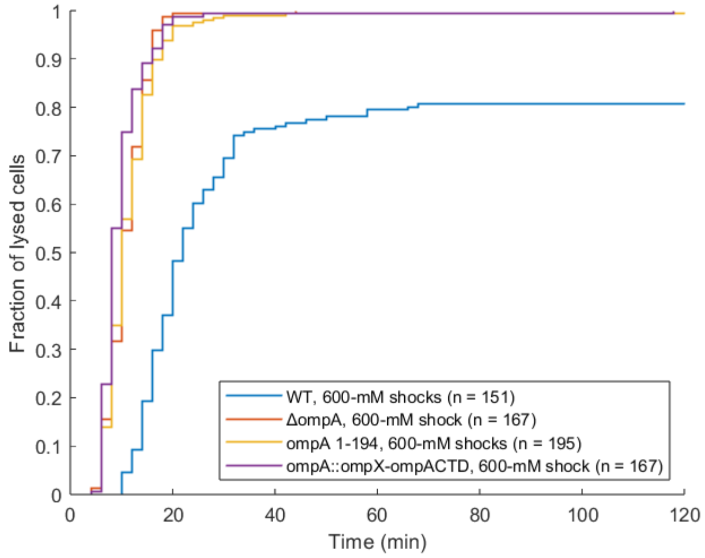

Fig. S6. Whole cell stiffness is assessed by resistance to repeated osmotic shocks. Wild type MG1655 cells survived many shocks, however Δ*ompA*, *ompA*$^{1\text{-}191}$ and *ompX-ompA*$^{192\text{-}346}$ cells lysed within 30 minutes.

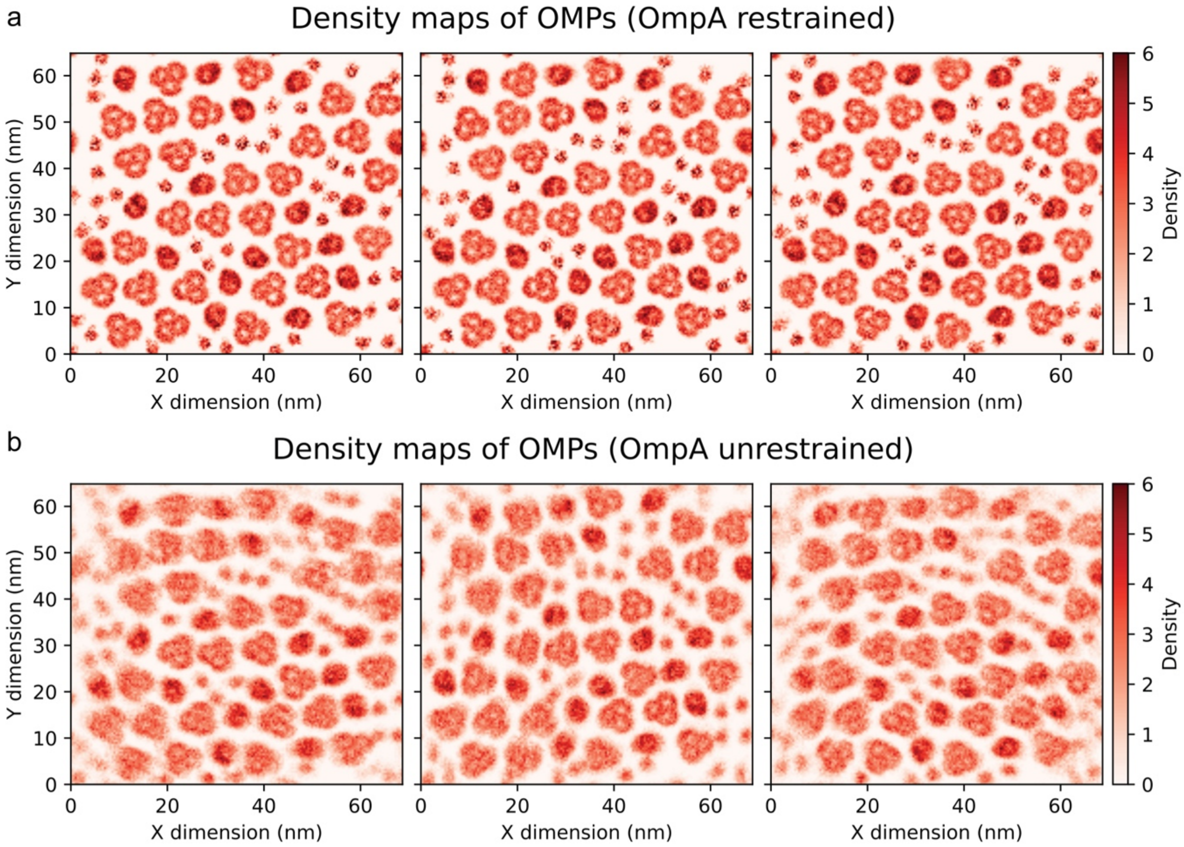

Fig. S7. Density maps from all three individual simulations. (a) OmpA restrained and **(b)** OmpA unrestrained cases show similar behaviour in each repeat.

Table S1. Strains used in this study.

Strain	Genotype	Source
GB001	MG1655	[2]
GB034	MG1655 lpp::kan	This study
GB077	MG1655 ΔompA	This study
GB078	MG1655 ΔompA	This study
GB079	MG1655 ΔompA	This study
GB165	MG1655 ompA 1-191	This study
GB166	MG1655 ompA 1-191	This study
GB167	MG1655 ompA 1-191	This study
GB197	MG1655 ΔompA::ompX-ompA192-346	This study
GB198	MG1655 ΔompA::ompX-ompA192-346	This study
GB199	MG1655 ΔompA::ompX-ompA192-346	This study
GB255	MG1655 ΔompA::ompX	This study
GB261	MG1655 ΔompA::lpp 1-35-ompA195-346	This study
GB262	MG1655 ΔompA::lpp 1-35-ompA195-346	This study
GB263	MG1655 ΔompA::lpp 1-35-ompA195-346	This study
GB266	MG1655 ompA D262A R277A	This study
GB267	MG1655 ompA D262A R277A	This study
GB268	MG1655 ompA D262A R277A	This study
GB275	MG1655 Δlpp	This study
GB276	MG1655 Δlpp	This study
GB277	MG1655 Δlpp	This study
GB370	MG1655 ΔompA::ompX-ompA D262A R277A 192-346	This study

Table S2. Plasmids used in this study.

Plasmid	Description	Source
pEXG2		[3]
pEXG2_tetAsacB	pEXG2 with tetA under control of the tetA/tetR promoter	This study
pCP20	Plasmid for expression of temperature sensitive flippase	[4]

Table S3. PCR primers used in this study.

Primer	Name	5' to 3' DNA sequences	Description	Source
GB86	OmpX(SacI)F	GAGGAGCTCgtAAAAAAATTGCATGTCTTTCAGCAC	See methods	[5]
GB87	OmpX-OmpAc_F	GTTGGTTACCGCTTCggtcagggcgaagca	See methods	[5]
GB88	OmpX-OmpAc_R	gccctgaccGAAGCGGTAACCAACAC	See methods	[5]
GB89	JLEo16-R-XbaI OmpACt	GGGtctagactaagcctgcggctgagttacaacg	See methods	[5]
GB90	JLEo20-F-Chrom-OmpX	atattcatggcgtattttggatgataacgaggcgcaaaaaATGAAAAAAATTGCATGTCTTTCAGCACTGG	See methods	[5]
GB91	JLEo22-R-Chrom-OmpACt	aaaaccccgcagcagcggggtttttctaccagacgagaacctaagcctgcggctgagttacaacg	See methods	[5]
GB96	ompA_delTetSB F	atattcatggcgtattttggatgataacgaggcgcaaaaacgtataatgtgtggaattagctt	See methods	[5]
GB97	ompA_delTetSB R	aaaaccccgcagcagcggggtttttctaccagacgagaacaagccgatctcggc	See methods	[5]
GB99	ompAc_delKm F	gcactcgtccggacaacggcatgctgagcctgggtgtttcctaccgtttcTAAattccggggatccgtcgacc	See methods	[5]
GB100	ompAc_delKm R	gcagcggggtttttctaccagacgagaacttaagcctgcggctgagttactgtaggctggagctgcttcg	See methods	[5]
GB106	extendSacTet_fwd	AAAGGCAAAAaaaaccccgcagcag	See methods	This study
GB107	extendSacTet_rev	CTCGTTGGAGATATTCATGGCGTATTTTGGATG	See methods	This study
GB116	ompASDRlamda_fwd	CTCGTTGGAGATATTCATGGCGTATTTTGGATGATAACGAGGCGCAAAAAatgAAAAAGACAGCTATCGCGATTGCAGTGGCAC	See methods	This study
GB117	ompASDRlamda_rev	AAAGGCAAAAAAAACCCCGCAGCAGCGGGGTTTTTCTACCAGACGAGAACttaAGCCTGCGGCTGAGTTACAACG	See methods	This study
GB125	lppchr_fwd	ATGAAAGCTACTAAACTGGTAC	See methods	This study
GB126	ompACTD_rev	TCAAGCCTGCGGCTG	See methods	This study
GB127	lpp_ompACTD_rev	ACTGGAGCTGCTTCAGTCTGAACGTCAGAAG	See methods	This study

GB128	lpp_ompACTD_fwd	GTTCAGACTGAAGCAGCTCCAGTAGTT	See methods	This study
GB129	lppOmpAfrag_fwd	CTCGTTGGAGATATTCATGGCGTATTTTGGATGATAACGAGGCGCAAAAAatgAAAGCTACTAAACTGGTACTG	See methods	This study
GB130	lpppalOmpAfrag_rev	AAAGGCAAAAAAAACCCCGCAGCAGCGGGGTTTTTCTACCAGACGAGAACtcaagcctgcggc	See methods	This study
GB135	ompXlamda_fwd	CTCGTTGGAGATATTCATGGCGTATTTTGGATGATAACGAGGCGCAAAAAATGAAAAAAATTGCATGTCTTTCAGC	See methods	This study
GB136	ompXlamda_rev	AAAGGCAAAAAAAACCCCGCAGCAGCGGGGTTTTTCTACCAGACGAGAACttaGAAGCGGTAACCAACAC	See methods	This study
ANC456	pEXGtet_VF	atcaatgatagagtgtcaacttcgagaattacgcgtaataagctagcttccataatcg	Amplify pEXG2 with ANC457 for Gibson assembly into pEXG2-tetAsacB	This study
ANC457	pEXGtet_VR	acccaagagggcatttttacaattcgttcaagccgagatcg	Amplify pEXG2 with ANC456 for Gibson assembly into pEXG2-tetAsacB	This study
ANC458	pEXGtet_IF	tattacgcgtaattctcgaagttgacactctatcattgatagagttattttaccac	Amplify tetA cassette with ANC459 for Gibson assembly into pEXG2_tetAsacB	This study
ANC459	pEXGtet_IR	atctcggcttgaacgaattgtaaaaatgccctcttgggttatcaagag	Amplify tetA cassette with ANC458 for Gibson assembly into pEXG2_tetAsacB	This study

Table S4. Other DNA oligos used in this study.

DNA oligo	5' to 3' DNA sequences	Description
FirstRecTforR	ACCGCATCGGTTCTGACGCTTACAACCAGGGTCTGTCCGAGCGCGCGGCTCAGTCTGTTGTTGATTACCTGATCTCCAAAGGTATCCCGG	Multiplex automated genome engineering oligo for *ompA*^{R277A} by CspRecT
SecondRecTforDR	CCTGGATCCGAAAGACGGTTCCGTAGTTGTTCTGGGTTACACCGCGCGCATCGGTTCTGACGCTTACAACCAGGGTCTGTCCGAGCGCGC	Multiplex automated genome engineering oligo for *ompA* $^{D262A/R277A}$ by CspRecT

SI References

1. The UniProt Consortium. UniProt: the Universal Protein Knowledgebase in 2023. *Nucleic Acids Res.* **51**, D523–D531 (2023).
2. Guyer, M. S., Reed, R. R., Steitz, J. A. & Low, K. B. Identification of a sex-factor-affinity site in E. coli as gamma delta. *Cold Spring Harb. Symp. Quant. Biol.* **45**, 135–140 (1981).
3. Hmelo, L. R. *et al.* Precision-engineering the Pseudomonas aeruginosa genome with two-step allelic exchange. *Nat. Protoc.* **10**, 1820–1841 (2015).
4. Cherepanov, P. P. & Wackernagel, W. Gene disruption in Escherichia coli: TcR and KmR cassettes with the option of Flp-catalyzed excision of the antibiotic-resistance determinant. *Gene* **158**, 9–14 (1995).
5. Dekoninck, K. *et al.* Defining the function of OmpA in the Rcs stress response. *eLife* **9**, e60861 (2020).
